# Combinational System of Lipid-Based Nanocarriers and Biodegradable Polymers for Wound Healing: An Updated Review

**DOI:** 10.3390/jfb14020115

**Published:** 2023-02-18

**Authors:** Bahareh Farasati Far, Mohammad Reza Naimi-Jamal, Meysam Sedaghat, Alireza Hoseini, Negar Mohammadi, Mahdi Bodaghi

**Affiliations:** 1 Research Laboratory of Green Organic Synthesis and Polymers, Department of Chemistry, Iran University of Science and Technology, Tehran 1684613114, Iran; 2 Advanced Materials Research Center, Materials Engineering Department, Najafabad Branch, Islamic Azad University, Najafabad 8514143131, Iran; 3 Department of Materials Engineering, Iran University of Science and Technology, Tehran 1684613114, Iran; 4 Department of Pharmaceutics, Faculty of Pharmacy, Ahvaz Jundishapur University of Medical Science, Ahvaz 6135733184, Iran; 5 Department of Engineering, School of Science and Technology, Nottingham Trent University, Nottingham NG11 8NS, UK

**Keywords:** biodegradable polymers, wound healing, lipid-based carriers, combinational systems

## Abstract

Skin wounds have imposed serious socioeconomic burdens on healthcare providers and patients. There are just more than 25,000 burn injury-related deaths reported each year. Conventional treatments do not often allow the re-establishment of the function of affected regions and structures, resulting in dehydration and wound infections. Many nanocarriers, such as lipid-based systems or biobased and biodegradable polymers and their associated platforms, are favorable in wound healing due to their ability to promote cell adhesion and migration, thus improving wound healing and reducing scarring. Hence, many researchers have focused on developing new wound dressings based on such compounds with desirable effects. However, when applied in wound healing, some problems occur, such as the high cost of public health, novel treatments emphasizing reduced healthcare costs, and increasing quality of treatment outcomes. The integrated hybrid systems of lipid-based nanocarriers (LNCs) and polymer-based systems can be promising as the solution for the above problems in the wound healing process. Furthermore, novel drug delivery systems showed more effective release of therapeutic agents, suitable mimicking of the physiological environment, and improvement in the function of the single system. This review highlights recent advances in lipid-based systems and the role of lipid-based carriers and biodegradable polymers in wound healing.

## 1. Introduction

The wound healing process starts with the activation of immune system cells, coagulation, and inflammatory responses in which all skin compartments are remodeled [1]. The wound healing process includes inflammation, proliferation, and remodeling (maturation) stages [2]. The inflammatory phase begins when various factors damage tissue, with bleeding in some cases. Thereupon, platelets adhere, become activated, and aggregate, which causes a clot made of fibrin. Forty-eight hours after the lesion first appears, the proliferation stage will begin [3]. When new blood vessel formation starts, numerous components are involved, such as new granulation tissue composed of fibroblasts and endothelial cells, which fill and cover the damaged tissue. Connective tissue granulation facilitates the formation of an extracellular matrix. The third phase of the wound healing process begins within 2–3 weeks later, following the onset of the lesion, and can extend for several months or more; this stage is known as remodeling or maturation. The best treatment approach for wounds has to be effective, highly stable, and inexpensive with minimal side effects [4]. Although many different researches studied wound healing, there are still many problems, such as non-healing wounds, surgical scars, and trauma [5]. For this purpose, novel therapeutic approaches are necessary for developing wound healing care to reduce healthcare expenses and increase treatment efficacy. Nanotechnology is one of the best approaches to wound healing care problems [6]. LNCs could be excellent candidates to improve previous therapeutic approaches, standardize care, and handle wounds [7]. LNCs benefit from good characteristics such as small size, high drug integration capacity, a large surface-to-volume ratio, and high reactivity. In addition, new lipid-based drug delivery systems can be tailored to improve drug release control and the skin repair rate [8]. Moreover, using lipid-based drug delivery systems is a promising approach for enhancing the poor permeability and bioavailability of poorly soluble medicines in water. Applying biocompatible polymers and drug-loaded systems associated with lipid-based wound therapies improve the effectiveness of wound therapies. In addition, experimental in vitro, ex vivo, and in vivo models are necessary to determine the efficacy of hybrid systems [9]. Biodegradable polymers can be designed for diverse drug-delivery applications. These polymers’ ability to break down into harmless monomers within the body allow them to be used for drug delivery systems. Many studies have highlighted the development of novel supra-molecular structures to transport genes and macromolecules [10,11]. Developing closed-loop drug delivery systems could be helped by discovering hydrogels that respond to a wide range of physical, chemical, and biological stimuli. Today, hydrogel dressings are the most common biodegradable polymer wound dressings. They have excellent properties such as being hypoallergenic, high moisture content, softness, and flexibility [12]. Biopolymer hydrogel-based wound dressings have become increasingly significant in modern wound healing because of their special features such as being easy to dispose and able to absorb moisture from exudates [13]. For example, wound dressing based on alginate forms is a very hydrophilic gel that can absorb fluid and prevent bacterial contamination. Furthermore, alginate dressings should not be used on dry or severely burned wounds, as they are not suitable for these types of wounds. However, they are suitable for moderate to high exudation wounds. [14]. Many studies focus on biodegradable synthetic polymers because there is no need to remove implants made of these materials from the patient’s body. Biodegradable polyesters are the most commonly applied materials in tissue engineering because they have excellent properties, including biocompatibility and control of the body’s inflammatory response [15,16]. This review describes a summary of the development of lipid-based drug delivery systems in the combination of biodegradable polymers, which emphasizes the connection between the physicochemical properties of these systems and their effect on the wound healing procedure.

## 2. Mechanism of Lipid-Based Drug Delivery Systems

**Figure 1:**
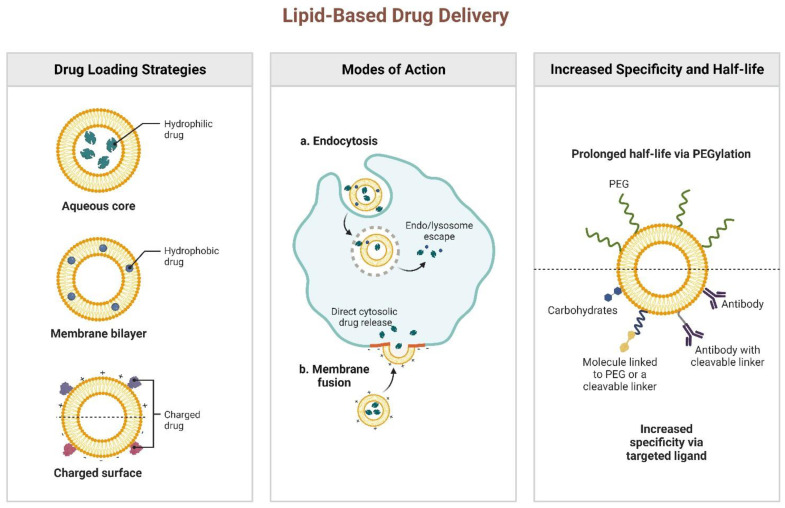
Structure, mechanism of internalization, and functionalization of liposomes in drug delivery systems. The liposomes are comprised of a lipid bilayer with hydrophilic heads and hydrophobic tails. The mechanism of internalization can occur through endocytosis or phagocytosis. The functionalization of liposomes can be improved by attaching targeting moieties or incorporating active agents.

Drug delivery methods based on nanosized lipids have shown significant efficacy for cutaneous and transdermal administration. They are made up of biocompatible lipids and are biodegradable, allowing for controlled release, targeted distribution, and drug protection. Multiple therapeutic substances, such as growth factors, genes, or cytokines, can be encapsulated with these drug delivery systems, which has been noted in the literature [17]. Intact skin is known to prevent the absorption of topical treatments. Even in injured skin, investigations have shown that drug molecules have low permeability, highlighting the need for innovative delivery mechanisms to ensure that drugs reach their intended target [18]. Lipid-based nano-drug delivery systems offer promising solutions to these problems. LNCs benefit skin wound healing and can transport drug molecules to the intended sites (Figure 1). They are divided into vesicular systems and LNPs [19]. Amphiphilic molecules, which have both a hydrophilic head and a non-hydrophilic tail, make up vesicular systems. Lipid vesicles have several valuable properties, such as their ability to bind to the corneous layer’s lipid matrix and to increase the number of drug molecules that penetrate deeper skin layers due to their size range of 40–800 nm [20]. It is interesting to note that the particle sizes of the vesicles affect how well the drugs penetrate the skin.

**Figure 2:**
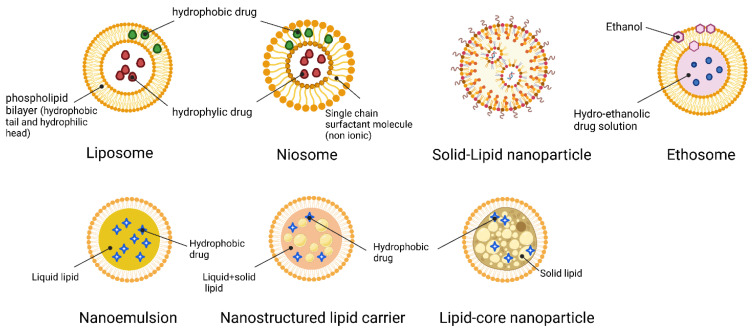
Different types of LNCs for hydrophilic/hydrophobic drug delivery.

Vesicles having a size of 70 nm have been found to deliver the most molecules [21]. More and more research is being performed on LNPs, which, according to recent discoveries, are preferable to vesicular systems because they can reach deeper layers of the epidermis [22]. LNPs also allow biocompatible components approved for pharmaceutical and cosmetic use, molecules with varied solubility, improved skin diffusion by combining physical and mechanical enhancers, targeted delivery, and lower systemic side effects. A subset of LNPs known as solid nanoparticles (SLNs) offers an alternative to more conventional lipid-based formulations such as liposomes, niosomes, and emulsions [23]. The next generation of LNPs is nanostructured lipid carriers (NLCs). A more complex lipid matrix in NLCs, including liquid lipids (such as: oils), generates changes in the structure of the SLNs, resulting in a less ordered crystalline arrangement that prevents drug leakage and supplies a higher drug load [24]. This contrasts with SLNs, which consist entirely of solid lipids and a perfectly smooth matrix. Factors such as the LNPs’ size also play a role in determining the distribution mechanism. Particles smaller than 10 nm can easily break through the stratum corneum of healthy human skin, while particles between 10 nm and 200 nm can enter the skin via the hair follicles [25,26]. Furthermore, the LNPs physiological lipid composition improves drug permeability through rearrangement and fluidization of the stratum corneum lipid matrix. As a result of the occlusion they create, water loss is reduced, which in turn results in more hydrated skin. The schematic structure of LNCs is represented in Figure 2.

### 2.1. Drug Delivery Methods Based on lNCs in the Wound Healing

The skin plays a significant role in maintaining body fluids, thermoregulation, and preventing microbial invasion. The pathophysiology of various skin wounds may necessitate unique approaches to healing. Acute burn wounds cause damage to the skin’s underlying tissues and the rest of the body’s systems because plasma leaks into the interstitial spaces. This can cause hypovolemic shock, which can be fatal, depending on how far the burn has progressed. The compromised skin barrier also heightens bacterial infection vulnerability [27]. Therefore, preventing infections and promoting re-epithelization to retain skin functionality is essential in treating burn wounds. Antimicrobial topical treatments have been the focus of most research, although their effectiveness is decreasing in the face of rising antibiotic resistance. Liposomes, transfersomes, ethosomes, and LNPs are a few examples of lipid-based delivery systems that have been investigated for their potential to combat infections and promote skin regeneration in burn wounds. Ethosomes are a type of vesicular drug delivery system that are composed of phospholipids, ethanol, and water. They are similar to liposomes in that they have a phospholipid bilayer but differ in that they also contain ethanol, allowing for enhanced skin penetration. Due to their high biocompatibility and ability to absorb numerous natural and partially synthetic compounds, LNPs are useful as topical carriers due to their high drug incorporation efficiency and versatility in synthesis. Their lipid structures give them properties that prevent water evaporation from the skin and keep it well hydrated. One of the most important applications of LNPs is their ability to accelerate wound healing and maintain hydration [28,29]. The maximal occlusion effect was shown to occur with particles that both were small in size and had a low melting point. When the underlying tissues are slashed by sharp-edged devices, as in a laceration, the first line of therapy is to clean the area and remove any foreign bodies or dead tissue. It is considered chronic when the repair process takes more than three months, which can be caused by several factors, including a protracted inflammatory response, ongoing infection, insufficient angiogenesis, or excess reactive oxygen species [30]. Venous insufficiency and diabetes are the leading causes of chronic wounds. Nanoparticle-containing dressings are being used in the treatment of chronic wounds because of their ability to provide a moist environment, ward against infection, and speed up the healing process. New treatments for chronic wounds have been made possible through advances in lipid-based nano-drug delivery systems, which have increased the half-time and bioavailability of the drugs while decreasing their cost, toxicity, and several applications. Multiple nanosized-lipid-based drug delivery systems have been developed to treat diabetic wounds, showing encouraging results when therapeutic compounds are used across the wound healing process. Since LNPs occlude the stratum corneum, they prevent trans epidermal water loss and keep the lesion moist, making them a promising treatment option for burn and chronic wounds [31]. It is also argued that NLCs are superior to vesicular systems as a nano-delivery mechanism. They assist in decreasing the frequency of medication administrations and the time it takes for wounds to heal due to their advantages, such as minimal toxicity, high drug-loading capacities, and sustained release over a long period. Infection, hematomas, and dehiscence are all complications that can arise from surgical wounds because of the heightened inflammatory reaction caused by suture materials. As a result, patients spend more time in hospitals, resulting in higher medical bills and a greater risk for complications [32]. Wounds of this type can be treated well with antibacterial advanced wound dressings [33]. Excisional wounds in rats were successfully treated with lipid-based nano-drug delivery devices [34]. In addition, excessive collagen production during the remodeling phase after surgical wounds can cause keloid or hypertrophic scarring [34]. The skin serves a vital purpose in protecting the body from harmful external influences, but it also blocks the absorption of topical medications. The stratum corneum, composed of corneocytes and a lipid-rich matrix, serves as the skin’s principal barrier (chain ceramides, free fatty acids, and cholesterol). It blocks the skin’s ability to take hydrophilic or macromolecular drugs [35]. As the stratum corneum is impenetrable for most drugs, a biochemical strategy is needed to transport both hydrophilic and hydrophobic therapeutic molecules into the skin, and lipid-based nano-drug delivery devices are one such method. An active topical chemical can take three routes through the skin: intracellular, intercellular, and trans follicular [36]. Liposomes use an intracellular delivery mechanism penetrating the skin and depositing medications in the epidermis for extended release. In addition, they shield the injury and keep the skin above it wet, which is beneficial to the healing process. However, elastic liposomes and transferosomes can permeate undamaged skin by squeezing through the intercellular sections of the corneous layer. Ethanol makes ethosomes pliable enough to fluidize through the lipids of the stratum corneum and transport their cargo below the corneous layer [37]. One recently published study shows that ethosomes can carry drugs via intercellular and transcellular channels [38]. However, nanoparticles may preferentially enter the skin via the trans follicular and intercellular pathways [39]. In addition, unlike the intracellular system, the trans follicular route can provide greater penetration of the active compounds [38]. To further improve the penetration of liposoluble compounds, SLNs show sustained interaction with the lipids of the corneous layer. However, NLCs are linked to an increased drug deposition in the skin, perhaps because of the liquid lipids present in the matrix. Additionally, LNPs’ attachment to the corneous layer induces regeneration of the skin’s lipid film, which allows them to keep the skin hydrated via the generation of a biofilm barrier at the stratum corneum’s surface. It is important to note that the formation of this lipid barrier aids in the absorption of the active ingredients into the skin [40]. The most important findings from this research are summarized in Table 1, and we will discuss the significance of hybrid systems of biodegradable polymers and LNPs in wound care in greater depth below.

Electrostatic interactions between the polar/ionogenic phospholipid head and the solvent, or the presence of non-polar lipid hydrocarbon moieties in the solvent, are the two main factors that govern the shape and size of LNCs [41]. Due to the presence of organic moieties on the surface of polymers (such as hydrocarbon moieties containing hydroxyl, carboxyl, amino, or amide functional groups), their surface characteristics may be quite comparable to those of some LNPs. Biological membranes (such as lung membranes and cell membranes) and, most importantly, lipid membranes interact with nanoparticles in different ways, and the surface features of the nanoparticles largely determine this.

The hydrophobic side chains of the polymers appeared to have a significant role in polymer partitioning within the membrane and the consequent membrane disruption, as was previously seen for polymeric nanoparticles.

Polyethylene glycol-coated (PEGylated) lipids are one type of modified lipids in which a lipid moiety is attached to the polymer core, and a polyethylene glycol (PEG) chain extends outward into the surrounding aqueous medium. The mechanism may involve the creation of a bilayer structure and its adhesion to the core, followed by the bilayer’s breakdown due to hydrophobic interaction between the polymer and the lipid chains. The hybrid creation has a positive thermodynamic outcome regarding hydrophobic, van der Waal, and electrostatic interactions [42].

PEGylated liposomes (in which PEG is covalently attached to the lipids) are far more resilient towards leakage than their non-PEGylated counterparts, and they maintain their integrity for more extended periods in live organisms [43].

Bolzinger et al. investigated the role of PEG in penetration mechanisms of PLA-based nano-formulations in the skin. Their results showed that PEGylated NPs increased surface wetting and interactions with skin lipids, enhancing drug absorption in intact skin [44].

#### 2.1.1. Liposomes

Liposomes as bilayer spherical nanocarriers have been noticeably used for topical administration, particularly wound treatment. Their significant properties, including biocompatibility and biodegradability, have led to a safe profile. Additionally, the amphiphilic structure facilitates the encapsulation of both hydrophilic and lipophilic pharmaceutical substances [72]. By taking advantage of biocompatible liposomes, the toxicity of potentially toxic components could decrease drastically. For example, silver ultra-small nano cluster liposomes show no cytotoxicity effect on umbilical vein endothelial cells in contrast to free sliver nano clusters and Ag NO_3_ that affect cellular viability dramatically [73]. Liposomes provide enhanced permeation through the skin due to their fluidity and alterations that they implement on stratum corneum arrangement [74]. Li et al. [75] designed an efficacious liposomal formulation from madecassoside that demonstrated a water-soluble substance’s amplified wound healing effect by complete wound exposure for 12 days [75]. Besides intriguing advantages, liposomes might represent inappropriate wound dressing owing to low viscosity, which makes the healing process longer and causes complications such as infections. To overcome this, composites containing different scaffolds such as sodium alginate [76], chitosan [77], and three-dimensional supports would be provided to achieve sustained and controlled wound management [78,79]. Raj Kumar et al. prepared collagen-based hydrogel as a scaffold to prolong the effect of liposomes loaded with vancomycin applied on MRSA-infected wounds [80]. Vancomycin with highly water-soluble characteristics has a weak topical absorption and, hence, would be eliminated readily. According to this experiment, the novel formulation manifested a controlled release of vancomycin even after a minimum of two fresh MRSA inoculations in both in vivo and in vitro studies. Zeta potential is a parameter that describes the electrical charge on a liposome’s surface that plays an essential role in the infiltration of liposomes’ preparations through lower layers of skin; consistent with the negative charge of skin lipids, cationic liposomes generally have a better penetration [81]. An attempt to improve growth factor permeation was made by J Uk Choi et al. They combined cationic elastic liposomes fused with growth factors consisting of PDGFA-A, EGF, IGF-I, then conjugated with hyaluronic acid [82].

Finally, these results showed the highest amount of diabetic wound constriction because of compounded flexible liposomes, which accelerate the re-epithelialization process. Moreover, they incorporated cationic liposomes and finally cooperated with the PLGA nanofibrous membrane through an electrospinning process. The final formulation represented a great synergistic vascularization and repair effect at the site of the wound, with enhanced differentiation [83]

Despite all merits and developments that were mentioned in this section, there are several disadvantages to be highlighted: (I) a decrease in chemical and physical stability over time, (II) complications following industrial manufacturing, as conventional methods of fabrication are impractical in large scales, (III) low permeation through deep layers of the skin [84]. For this reason, further studies are needed to concentrate on overcoming these challenges in clinical administration due to their worthwhile efficacies in wound treatment.

#### 2.1.2. Niosomes

**Figure 3:**
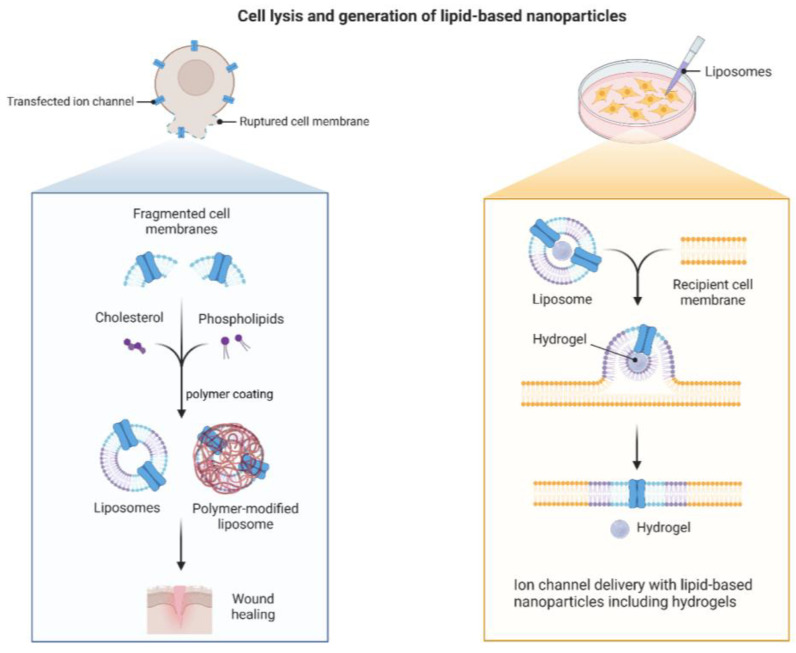
Cell lysis and generation of lipid-base nanoparticles in wound healing tissue engineering.

Niosomes are defined as self-assembled, bilayered vesicles made from non-ionic surfactants in the presence of cholesterol. Niosomal formulations are biocompatible, exhibit non-immunogenic properties, and can encapsulate both hydrophilic and hydrophobic active compounds. These lipid-based carriers have also shown better penetrating potential and stability than liposomes [85,86]. Studies support that the combination of niosomes and polymeric systems possesses proper antibacterial activity and promising characteristics [87,88]. Niosomes and polymers do not inherently possess antibacterial properties, but they can act as a carrier for active substances that have antibacterial properties. Hydrogel formulations with 3D structures and biocompatibility can keep the wound environment at a lower temperature and preserve the moisture in the area, promoting cell migration and proliferation and, thus, improving wound healing (Figure 3) [89]. Recently, a new formulation was developed and characterized using Aloe Vera extract-loaded niosomes embedded in alginate/gelatin hydrogel for treating wounds. Results demonstrated that niosomes could efficiently encapsulate Aloe Vera extract, which is known for its stimulating effects in the proliferation of fibroblasts. The prepared hybrid system also sustains the therapeutic agent’s release [90]. Moxifloxacin has shown excellent potential against a wide range of bacteria which may help wound healing and makes it an option for treating wound infection. Sohrabi et al. have taken advantage of both chitosan-based hydrogels, which can make a bio adhesive matrix for local drug delivery, and niosomes to sustain the release of moxifloxacin. This combinational system showed sufficient bio adhesiveness, sustained drug release, and great anti-*S. Aureus* activity in an in vitro investigation [91]. In another study, simvastatin was selected as a drug model with antibacterial effects. The lipophilic nature of this statin, and, thus, low water solubility, may have affected its application as an antibacterial agent [86]. Niosomes as novel carriers could encapsulate simvastatin and address this problem. Niosomes were then embedded in chitosan-based gels, and their antibacterial effects were assessed against two of the most commonly presented bacteria strains known for causing skin infections, *E. Coli* and *S. Aureus.* Results indicated that this niosomal formulation could entrap highly hydrophobic drugs and better fight against *E. coli* and *S. Aureus* [92]. The impact of the niosomal-sodium carboxymethyl cellulose gel was also assessed in an in vivo experiment using full-thickness skin wounds in rats. Niosomes were loaded with methylene blue as a hydrophilic agent with an antioxidant property capable of decreasing reactive oxygen species production [87].

Treatment with niosomal formulation caused a reduction in malondialdehyde while increasing the level of superoxide dismutase. The histopathology reports showed that more fibroblasts and thicker collagen fibers were observed in the rats administered with niosomal gel [87]. Niosomes were also combined with cross-linked chitosan nanofibrous membranes for wound healing. Cefazoline-loaded niosomes were fabricated using thin-film hydration and electrospray on the surface of the nanofiber. Results confirmed the anti-bacterial and angiogenic effect of this new hybrid formulation making it a good choice for skin regeneration [93]. Niosomes hold great promise in topical application and wound healing. Moreover, its good permeability may be attributed to its ionic part, which can interrupt cellular connectivity [94]. Based on the evidence, hydrogel is the best pharmaceutical formulation for niosomes to be blended [95].

#### 2.1.3. Solid Lipid Nanoparticles (SLNs)

SLNs are known as versatile nanosized carriers due to their noticeable capability to encapsulate a broad range of semi-synthetic and natural medications regardless of their lipophilicity or hydrophilicity properties [96]. SLNs provide an advanced dispersion of solid physiological lipids (0.1–30%) in water or aqueous solutions and are stabilized via surfactants (0.5–5%) including Poloxamer 188, Lecithin, and Tween 80 [97,98]. They are generally biocompatible with low toxicity grades, showing improved stability and bioavailability of the incorporated drugs [99,100]. Morphologically, most SLNs are spherical with limited particle size distribution and possess a large surface area. Furthermore, it is possible to manufacture them on an industrial scale. Furthermore, they are autoclavable so that they can be sterilized easily [101].

Moreover, dermal permeation characteristics and controlled drug delivery are noticeable properties for topical administration [102]. Accordingly, much research has been conducted to investigate the efficacy of SLNs formulation for wound management. For example, Curcumin, a poor water soluble and photosensitive herbal extract, was encapsulated in a combinational system of SLNs and hydrogel, with an encapsulation efficiency of 77% and a polydispersity index (PDI) of 0.143. This increased solubility without any usage of organic solvent [103]. Apart from this, the aforementioned formulation augmented curcumin’s anti-inflammatory effect, which was demonstrated by a distinct decrease in TNF-α and great vascularization owing to VEGF and CD31+ cells rising at the wound site. In vitro drug release depicts a prolonged curcumin release and a zero kinetic; indeed, just 38% of curcumin was released. Lastly, *S. Aureus* biofilm eradication by SLN was obtained. Thus, these developments accelerated wound recovery [59]. Chamomile oil can be named as a further example of facilitating the delivery of herbal extracts by SLNs. It presents a noticeable vulnerability to moisture and oxygen. Encapsulation in SLN vehicles improved its healing properties and resulted in acceptable wound remedies through regulating inflammatory factors [69]. Another example is loading Hibiscus Rosa Sinesis extract on SLNs reported by Vijay et al. It enhanced antioxidant effects and gave rise to absolute wound size decline during 16 days on in vivo wound models [104].

Regarding studies emphasizing amplifying antimicrobial activity, an SLN combination with neomycin showed excellent antibiotic activity for mucosal wounds [105]. Moreover, integrating neomycin SLNs in a gel based on Kolliphor-407 P contributed to a consistent structure that sustained release (84.87%) for 24 hours. This novel formulation also promoted neomycin bioavailability because of successful mucosal membrane permeation. Furthermore, it could preserve its efficacious properties after one month of storage at 25 °C, enhancing its effect on wound healing [54]. Simvastatin (SIM), an HMG-Co reductase inhibitor known for its cholesterol-lowering effect, had been encapsulated in self-gelling SLNs to evaluate its wound-repairing activity. Applying SIM-loaded SLN hydrogel on intact rabbit skin exhibited no irritation; additionally, administering 1 mg SIM formulation on wound models led to complete wound closure owing to comparable collagen deposition and regenerative effect [106]. Another unconventional SLNs hydrogel was designed by loading Valsartan as an angiotensin II blocker to exploit its demonstrated efficacy on diabetic ulcers. The formulation with high entrapment efficacy and an extended release manifested a significant diabetic foot wound shrinking including a notable reduction in COX-2, MMP, and NF-κB and a significant disruption to filming formation of both gram-positive and negative bacteria [107]. Opioids induce keratinocyte migration caused by increased nitric oxide production. Therefore, a promising SLN formulation of morphine was developed to administer on a 3D wound model. SLNs helped to overcome insufficient morphine concentration at the wound site, which is attributable to a long-term and controlled drug release.

Consequently, applying this formulation speeded wound contraction for four days, along with negligible irritation and cytotoxicity [108]. However, SLNs with significant potential to improve the condition of wounds because of simplifying cargo delivery might encounter some challenges. Firstly, the drug expulsion phenomenon, shown throughout the cooling process or promptly after preparation and storage, is mainly attributed to the tremendous crystalline lattice of solid lipids (especially with high purity) [109]. Secondly, the difficulty in epidermal targeting could be solved by implementing some targeting molecules on SLNs’ surfaces in future research on wounds.

#### 2.1.4. Lipid Core Nanocapsules (LCNs)

Several treatment techniques have been recently developed to supplement the structural properties of bio-degradable polymers, such as providing a physical support for tissue regeneration with antibacterial or wound healing-promoting qualities. Lipid-core nanocapsules have advantageous properties such as combination with multiple biopolymers and significant biodegradability; hence, they are used in drug delivery systems, implant coatings, etc. Their main advantages are sustained release, drug selectivity and effectiveness, drug bioavailability improvement, and drug toxicity alleviation.

Masci et al. analyzed loading curcumin into LNPs and embedding these particles inside collagen scaffolds by a double-encapsulation strategy. They observed that the distribution of nanoparticles among the collagen fibers was uniform, and no change was observed in the collagen structure [70]. Furthermore, fiber coatings with LNPs reduced the degradation rate, which made the structure last longer.

In other research, Carletto et al. studied ursolic acid-loaded LCNs to accelerate wound healing induced by estrogen deprivation. Their work indicated that LCN containing UA (Ursolic Acid) might prevent menopause-related skin damage. In vivo tests utilizing this compound as a dermal biostimulant were meticulously developed to confirm this hypothesis [46]. In the in vivo study, treatment with LCN-UA led to a quicker wound contraction and a reduced inflammatory response for wound healing. In addition to stimulating the angiogenic process, enhanced cutaneous collagen production occurred. Nanocapsules with a lipid-core and a load of UA help treat skin changes caused by a decline in estrogen during the menopause period. Thus, LCN-UA could be a suitable carrier because they reduce the amount of estrogen, which prevents skin aging, enhancing wound and skin healing quality [46].

Another study investigated the effect of an antibacterial polymer-lipid encapsulation matrix as an implant-coating. This coating, containing doxycycline-controlled release, protects from infection when encountering a doxycycline-resistant strain [45]. A combination of oil and nanocapsules can be helpful in the wound-healing process. Another study investigated the role of LNC containing Caryocar brasiliense Cambess in wound healing. The results indicated that this formulation is the best alternative for release systems, avoiding losses and complications related to cutaneous lesions in wound [68].

Research on nanocapsules based on lipids is developing as an alternative technique for most biological applications. This review aimed to study the biomedical advances in LCNs as a drug delivery mechanism. LCNs as drug delivery systems can improve the bioavailability of compounds and achieve sustained release of drugs with target-specific delivery, which will overcome the limitations of conventional methods used in wound healing. Polymeric biomaterials and LCNs are the best combinations for drug delivery systems because they lead to high efficiency in the controlled release of the encapsulated compound and have been the focus of various studies. LCNs protect the drug inside from failure or damage caused by the biological environment. Moreover, LCN reduces problems related to cutaneous diseases.

#### 2.1.5. Nanostructured Lipid Carriers (NLCs)

NLCs are novel formulations in second-generation lipid-based carriers composed of lipids, surfactants, and co-surfactants. Biocompatibility, low toxicity, and improved drug stability and loading capacity make them eminent candidates for drug delivery [110,111]. Studies have utilized NLCs in combination with biodegradable polymers [49] for wound healing which will be discussed in this section. Synthetic polymer-based gels are the most common formulations used to increase viscosity and residence time in favor of cutaneous treatment [112]. The hybrid NLC-gel formulas are easy to fabricate, stable, and suitable to be topically applied, which may guarantee better wound healing. In a pre-clinical study, recombinant human thrombomodulin (rhTM) was selected as an angiogenesis factor in improving diabetic wound healing. rhTM was successfully loaded in NLC with a reported encapsulation efficiency above 92%. Then the drug-loaded carrier was combined with a carbopol-based gel, which induced angiogenesis and re-epithelization [49]. Chitosan is considered a biodegradable natural polymer with a suitable hemostatic effect and can be used in wound healing scaffolds, while its brittleness has limited its application. Blending chitosan with other polymers, including hyaluronic acid (HA), can address this disadvantage [113]. A well-characterized chitosan-HA hybrid scaffold was prepared, and NLCs containing andrographolide were added to the scaffold mentioned. This novel hybrid formulation achieved controlled drug release, favorable swelling ratio, and porosity. Furthermore, in vivo observation indicated that the group treated with this hybrid drug delivery system showed enhanced histological progress and reduced scar formation [55]. In another study, Mathew et al. chose collagen polymer due to its 3D structure and satisfactory properties in wound healing. [114]. In this work, microstructure scaffolds were designed and combined with NLCs loaded with siRNA. The addition of NLCs did not alter the characteristics of the platform, including the swelling ratio, but the prolonged release was observed in this hybrid formulation. Moreover, in vitro reports implied the downregulation of one of the leading players in cellular processes, namely the extracellular signal-regulated kinase 1 (ERK-1) protein [115].

NLCs can also be incorporated into nanofibrous mats. PLGA-based nanofibers were loaded with Aloe Vera extract, known for its antibacterial and anti-inflammatory properties. NLCs were then added to the drug-loaded membrane to work as a lipid component and lessen adhesion to the application site, thus avoiding pain and injuries during wound dressing change. Samples with NLCs demonstrated higher water uptake and tensile strength. Factors including porosity and water vapor transmission rate were almost identical in samples with or without NLCs [50]. In recent years, NLCs have grabbed attention as a safe drug delivery system in topical applications due to their ability to release active agents in a controlled manner and local delivery. Furthermore, characteristics of NLCs may lead to enhanced healing rates [116]. Hydrogel, nanofiber, and scaffolds are some polymer-based systems suitable for combining NLCs to promote wound healing [117].

#### 2.1.6. Miscellaneous Lipid Nanocarriers

One of the most promising applications of LNCs in wound healing is their ability to deliver growth factors and other biomolecules directly to the wound site. Growth factors, such as platelet-derived growth factor (PDGF) and transforming growth factor-beta (TGF-β), are known to play a crucial role in the wound healing process by promoting cell proliferation and differentiation and stimulating extracellular matrix remodeling. LNCs, such as liposomes, can effectively encapsulate and protect these growth factors from degradation and allow for targeted delivery to the wound site. In addition, LNCs can also be functionalized with specific peptides or antibodies to enhance their targeting abilities further. Studies have shown that using LNCs loaded with growth factors can significantly improve wound healing outcomes, including increased re-epithelialization and collagen deposition [118]. Lipids such as phospholipids, steroids, oils, and fatty acids have been used extensively in manufacturing LNPs, such as liposomes, niosomes, SLNs, and NCLs. Some of their characteristics include nanoscale, lipophilic nature, viscoelastic properties, and functions of lipid nanocarriers, allowing for increased drug loading, stability, enhanced penetration through the stratum corneum and a progressive release of the drug in the targeted skin layers. Due to their biocompatibility, low toxicity, and resemblance to skin components, LNPs have garnered considerable attention for topical distribution. They provide advantages such as increased skin penetration and retention, controlled release, reduced dose, a more significant pharmacological effect, and patient compliance [119,120]. Previous studies indicated that LNCs are the best candidates for drug delivery systems studies because of their small particulate size, stability, enhanced oral absorption, excellent bioavailability, easy permeation across intestinal membranes, and protection against harsh gastrointestinal conditions. Applying polymers’ combinations and different strategies improves the stability of the hydrophilic drug in the lipophilic core. In addition, high entrapment efficiency and easy scalability were necessary to develop an inexpensive, non-invasive drug delivery system. Applying polymers’ combinations and different strategies improves the stability of the hydrophilic drug in the lipophilic core. In addition, high entrapment efficiency and easy scalability were necessary to develop an inexpensive, non-invasive drug delivery system [121,122].

Cano et al. examined particle tracking (PT) data processing by developing a unique screening method for particle trajectories. These data have been compiled into a program accessible to the public to facilitate the accurate study of PT data. They have then investigated the limits of this traditional technique using model polystyrene nanoparticles as a control and oral LNCs. Finally, the derived information from their research was compiled into an accessible and user-friendly PT analysis program, which contributed to the logical design and development of nanocarriers for drug delivery [118].

## 3. Biodegradable and Nondegradable Polymers for Wound Healing

Biodegradable polymers and synthetic polymers, such as wound dressing, have been studied in many types of research (Table 2) [123]. Some novel approaches have been developed to create three-dimensional scaffolds based on biomaterials. Such scaffolds have advantages for tissue engineering, such as promoting cell adhesion, proliferation, migration, and differentiation [124]. Many natural and synthetic polymers produce scaffolds in skin tissue engineering [125,126,127]. Scientists produced many biodegradable polymers to transport pharmaceuticals, macromolecules, cells, and enzymes into the body [34]. Biodegradation can be caused by enzymes, chemicals, or microorganisms, depending on the circumstances. Polyesters are one of the most extensively studied polymers for drug administration—for example, poly glycolic acid (PGA), poly lactic acid (PLA), and their copolymers poly lactic-co-glycolic acid (PLGA) [128].

### 3.1. The 3D-Printed Hydrogels 

Wang et al. fabricated chitosan methacrylate wound dressings with a 3DP technique. They showed the application of 3DP to fabricate wound dressings with customizable shapes and drug delivery systems according to the patient’s requirements. Results demonstrated a tunable release of drugs from the wound dressing and the precise and tunable loading of multiple drugs into the wound dressing. Moreover, it illustrated the capacity of 3DP to fabric customizable wound dressings in terms of shape, size, and composition. Shavandi et al. fabricated marine-based hydrogels via 3DP. The 3D printable hydrogels exhibited superior toughness, moldability, flexibility, and self-healing capacity [148].

Gutierrez developed antimicrobial alginate/bacterial-cellulose hydrogels with 3DP technology. Their results showed antimicrobial alginate-based 3D-printed materials using high printability inks for potential applications in tissue engineering and regenerative medicine [149].

Fatourosa et al. evaluated the physicomechanical properties of 3D printable algi-nate-methylcellulose inks for wound healing applications. This hydrogel had adequate water and water vapor sorption capacity, suitable antibacterial and antibiofilm activity, good biocompatibility on human dermal fibroblasts, and stimulating cell growth. Hence, it can be useful to wound management applications [150].

Santos et al. developed an antibacterial and biocompatible patch of a Manuka-Gelatin-based 3DP technology for wound healing applications. They showed that the 3D manuka-gelatin patches have properties suitable for wound healing and skin regeneration applications [151].

Alvarez et al. developed self-healing hyaluronic acid/chitosan poly complex hydrogels through a 3DP technique for drug delivery applications. This hydrogel demonstrated self-healing properties, good biodegradability, biocompatibility, and sustainable release. Hence, it can be used as 3D biodegradable scaffolds and soft implants for tissue engineering applications and drug delivery systems [152].

Liu et al. developed a multifunctional porous gelma/xanthan gum-based dressing via 3DP technique. Results demonstrated that the 3D-printed dressings had good antibacterial activity, suitable rheological properties, and accelerated wound healing. The wound dressings that were fabricated promote wound closure and shorten the closure time. In addition, 3DP technology and freeze-drying enhanced the swelling properties of dressings. The 3D-printed wound dressing accelerated wound healing and exhibited high antibacterial activity for preventing infection [153].

Many researchers are interested in 3D-printed hydrogels for wound healing applications. The 3DP technology has many benefits, such as controlling shape, size, and composition. Studied 3D-printed hydrogels had good mechanical properties such as toughness, moldability, flexibility; suitable rheological properties; and good biological properties such as biodegradability, biocompatibility on human dermal fibroblasts, stimulating cell growth, self-healing capacity potential applications in tissue engineering and regeneration, making them useful for wound management applications. Furthermore, hydrogel-based inks have been demonstrated to be a suitable vehicle for delivering the drug, and 3D-printed hydrogels can be used as scaffolds and soft implants for tissue engineering applications and drug delivery systems. They have good antibacterial activity, accelerate wound healing promote wound closure, and shorten the closure time.

### 3.2. Lipid-Based Hydrogels

Hydrogels are an important class of polymers and have excellent properties such as high biocompatibility, hydrophilicity, and a three-dimensional porous structure that matches the extracellular matrix, extensively explored as wound dressings in skin tissue engineering applications. However, their popularity is partly marred due to the maceration of adjacent skin caused by exudate retention, the requirement of secondary dressing, poor mechanical properties, and easy dehydration if kept uncovered [154]. Recently, lipid-based hydrogel received the attention of researchers. These hydrogels provided excellent conditions for the wound healing and drug delivery systems. SLNs have been playing a key role because the maintenance of many physiological processes in the body enhances the dissolution and bioavailability of oral drugs, and they offer high incorporation efficiency, an absence of biotoxicity as a carrier, better control over the drug release rate, and targeted delivery in the wound healing process.

El-ezz et al. prepared SLN-based hydrogel for wound healing. The hydrogel complex efficiently promotes various wound healing phases and could be a promising nanoformulation for cutaneous wound healing acceleration [155]. In another study, Fatima et al. fabricated SLN gels for diabetic wound healing. Its topical application effectively accelerates wound healing in diabetic conditions by sustaining the drug release for a prolonged period. The complete closure of the wound (100%) takes place within four weeks of gel application. They concluded that the gel showed collagen growth, promoting hemostasis and ingrowth of skin fibroblasts, endothelial cells, and keratinocytes. The gel is a promising topical dosage form for the effective treatment and management of diabetic wounds [156]. Din et al. evaluated the bioavailability and safety of cilostazol-loaded SLNs. Cilostazol-loaded SLNs showed a loss of crystallinity, excellent compatibility, optimal release, and enhanced bioavailability [157]. In another study, Gupta et al. established self-gelling SLN hydrogel as a wound dressing. Histology analytics showed the rate of wound healing was monitored along with the oxidative stress catalase. However, it will be appropriate to conduct more detailed studies such as monitoring various molecular markers of inflammation, healing, and angiogenesis as well as various genetic markers to elaborately establish the mechanism of action. Apart from appropriate safety, efficacy, and patient compliance, it will also be worthwhile and of scientific interest to study the structural aspects of the present formulation in depth and develop a more precise insight and understanding of its mechanical properties and sub-atomic structure [106]. Ahmed et al. developed SLN hydrogels for wound healing applications. Hydrogels loaded with argan oil and NPs demonstrated high encapsulation efficiency, better retention time, increased permeability, sustained in vitro drug release, and significantly enhanced bioavailability [158]. Asdaq et al. prepared a SLN-based hydrogel. This hydrogel improved antifungal therapy’s safety, affordability, and tolerance [159].

Singh et al. created SLN-based hydrogel. SLNs minimized the access of drugs to systemic circulation due to skin targeting and localization of the drug, thus, avoiding the systemic side effects. The research findings indicated that SLN-based hydrogel could serve as a promising carrier for topical drug delivery in clinical medicine [160].

Fran et al. developed and evaluated LNC-loaded tacrolimus. Their results showed that Tacrolimus loaded in lipid-core showed a sustainable release profile following the biexponential release model. Nanostructured lipid carriers (NLCs) are a highly promising drug-delivery system for the topical treatment of autoimmune diseases, as demonstrated in [161]. Deng et al. developed a polymeric hydrogel with a homogenous and porous structure with elliptical pores of non-uniform diameters. Results showed excellent biodegradability and biocompatibility. Moreoever, its the best candidate for long-lasting local anesthetics development without causing significant toxicity [162].

Ruiz et al. designed a dual NLCs/hydrogel system delivery for topical skin applications. This hydrogel provided a non-cytotoxic moderate antimigration/proliferation effect on dermal cell lines [163]. Tyagi et al. prepared a nanostructured lipid carrier hydrogel. NLCs facilitated drugs to accumulate and saturate in the epidermis and dermis, transporting them across the skin layers to confer therapeutic effects at diseased sites. Their study suggested the use of NLCs as second-generation colloidal nanoparticles to develop a delivery option for treating systemic inflammatory diseases [164].

Combining hydrogel with a lipid-based carrier, such as liposomes, can increase controlled drug release. Liposomes can overcome the limitation of rapid drug release of a hydrogel; therefore, combining these two delivery systems is a promising approach to achieve controlled dermal drug delivery and an effective wound healing process. Hemmingsen et al. developed a liposomes-in-chitosan hydrogel for wound therapy applications. They observed the anti-inflammatory effects of the hydrogel, an important feature considering wound therapy [165]. The anti-inflammatory properties of the hydrogel may be attributed to a combination of factors. The chitosan component of the hydrogel has been shown to have anti-inflammatory effects, potentially due to its ability to modulate the immune response. Liposomes have also been reported to have anti-inflammatory properties, likely due to their ability to encapsulate and deliver anti-inflammatory compounds. The hydrogel also contains chlorhexidine as an active substance, which has been reported to have anti-inflammatory properties. The combination of these different components in the hydrogel may synergize to enhance the overall anti-inflammatory effects of the hydrogel. Ternullo et al. developed a wound dressing comprising curcumin-in-liposomes-in-chitosan hydrogels. This hydrogel can be a good candidate for the sustained skin penetration of curcumin. The curcumin-in-liposomes-in-chitosan hydrogel developed by Ternullo et al. has been reported to enhance the skin penetration of curcumin compared to other delivery systems, such as conventional hydrogels that have been studied. However, it is important to note that this statement should be further qualified by comparing the curcumin-in-liposomes-in-chitosan hydrogel to other similar hydrogel systems and delivery methods in the literature.

Additionally, it should be mentioned that the sustained skin penetration of curcumin is not only due to the composition of the hydrogel but also the properties of the curcumin-liposomes and chitosan that lead to the sustained release of the active ingredient [166]. Pooprommin et al., developed an alginate/pectin dressing with niosomal mangosteen to enhance wound healing. This hybrid system demonstrated a high-water absorption rate, which served as an effective barrier against bacterial penetration. The advantage of combining niosomes with polymers in the alginate/pectin dressing with niosomal mangosteen is that it allows for the targeted delivery of the antibacterial compound, mangosteen to the wound site. Using niosomes as a delivery vehicle enables the controlled release of the mangosteen, which can improve the efficacy of the dressing in promoting wound healing. Additionally, the combination of alginate and pectin in the dressing provides a high water absorption rate, which can help maintain a moist wound environment that is conducive to healing. Overall, the combination of niosomes and polymers in this hybrid system allows for the synergistic effects of targeted delivery of a biologically active compound and maintaining a moist wound environment. Therefore, it can be a promising candidate for wound dressing applications [167].

## 4. The 3D-Printed Polymer–Lipid Composites

Today, 3DP has good potential in biomedical science. Numerous studies have studied polymer- and lipid-based applications.

Boyd et al. developed tablets that are a polymer–lipid-hybrid via 3D-Printed technology. Therefore this study presents opportunities to develop new dose forms with advantages in a polypharmacy context. Furthermore, it demonstrates a 3D-printed biodegradable tablet to deliver bespoke and combined therapies in a single multi-compartment tablet. The versatility of these 3D-printed tablets has the potential to circumvent challenges associated with the adherence and safety of polypharmacy, together with the 3DP of lipophilic drugs [168]. Additionally, the study reports that the versatility of these 3D-printed tablets has the potential to circumvent challenges associated with the adherence and safety of polypharmacy, together with those related to the 3DP of lipophilic drugs.

Behzadi et al. fabricated filament-based 3DP of dosage forms using lipid-based excipients. The produced filaments lacked defects and revealed much lower porosity, higher toughness, and better transportability within the 3D-printer than those produced by conventional solid feeding. The selection of lipid-based excipients paired with process know-how can extend the number of printable excipients, which has been limited to specific polymers. Future studies will focus on the solid-state stability of lipid-based 3D-printed formulations and the incorporation of potential drug candidates and, thus, explore the possibilities for innovative drug delivery systems [169].

Simon et al. developed novel ethylene vinyl acetate–lipid blends with lipid contents of up to 90% in order to improve the mechanical properties of lipids using 3DP technology. Hence, ethylene vinyl acetate and lipids were not suitable for 3DP on their own because they showed properties on the extremes that make them either too flexible and soft or too rigid and brittle. The 3D-printed tablets also measured dimensions close to the design values, which is important for accurate drug loading. Since the analysis of the 3D printed tablets suggest a role of viscosity of the blends in the final print quality, a study of the rheological behavior of the blends would assist in better understanding their effects on 3DP more generally. Future studies will address the impact of the incorporation of drugs into these printable compositions and drug dissolution and release in physiological conditions [170].

In another study, Beck et al. created an original platform based on 3D-printed nanomedicines to develop solid nanomedicines from liquid aqueous nanocapsule suspensions. This platform helped to develop novel, oral, customized nanomedicines for different applications, such as improving the bioavailability of active pharmaceutical ingredients or targeting them to specific organs and tissues [171].

Homan et al. fabricated and evaluated the effect of SLNs on a 3D-printed human auricular model. They concluded that the time for cells to grow on 3D printed models is critical because these models could lose their integrity. Their complex increased the proliferation of cells on a PLA auricular model and protected them from toxicity [172]. Barthélémy et al. prepared a nucleotide lipid-based hydrogel for ink fabrication. This polymer had suitable mechanical and biological properties for biofabrication applications. This approach was a powerful strategy for biofabrication, but further studies have been performed to investigate in vivo behavior [173].

Overall, 3DP is the best approach to fabricate biodegradable tablets to deliver bespoke and combined therapies; 3DP can overcome challenges such as poorly water-soluble drugs. The 3DP technique could develop solid self-micro emulsifying drug delivery systems with geometrical flexibility and complexity suitable for developing personalized lipid-based dosage forms, and printed tablets disintegrated rapidly and are well-defined in size with high mass uniformity and dose accuracy. Semi-solid extrusion can produce solid lipid tablets as filaments, thereby combining the advantages of lipid-based excipients as an established formulation strategy for poorly water-soluble drugs with a 3DP technology for solidification and flexible production, printing in hospital pharmacies of highly potent, poorly water-soluble drugs, for which flexible dose adjustments may be needed for patient groups such as children and the elderly.

## 5. Discussion and Future Perspective

The combined use of LNCs and bio-degradable polymers for wound healing has had increased interest in recent years. LNCs, such as liposomes and niosomes, have been shown to effectively deliver drugs to wound sites, while bio-degradable polymers, such as polylactic acid and polyethylene glycol, have been used as scaffolds to promote tissue regeneration. Together, these two systems have the potential to provide a synergistic effect for wound healing. LNCs have several advantages for wound healing. They are biocompatible and can encapsulate a wide range of drugs, including hydrophilic and hydrophobic compounds. They also have a high drug-loading capacity and can protect drugs from degradation in the body. In addition, they can target specific cells and tissues, such as macrophages, which play a key role in wound healing.

On the other hand, bio-degradable polymers provide physical support for tissue regeneration and can also release drugs over an extended period. This sustained drug release can help maintain an optimal therapeutic concentration at the wound site and reduce the need for frequent drug administration. The sustained release of drugs from the polymers can also help to maintain an optimal therapeutic concentration at the wound site.

In addition to these studies, combinational systems have also been developed for wound healing applications in human. LNCs and bio-degradable polymers have been used in wound dressings to deliver drugs and promote tissue regeneration.

Despite the promising results of combinational systems of LNCs and bio-degradable polymers for wound healing, some challenges still need to be addressed. One of the major challenges is the lack of a standardized method for evaluating the effectiveness of these systems. This makes it difficult to compare the results of different studies and to determine the optimal conditions for their use. Another challenge is the lack of long-term data on the safety and efficacy of these systems. More studies are needed to confirm the safety and efficacy of these systems in humans and determine the optimal dosage and duration of treatment.

In conclusion, combinational systems of LNCs and bio-degradable polymers have the potential to enhance the overall wound-healing process. LNCs can effectively deliver drugs to the wound site, while bio-degradable polymers can provide physical support for tissue regeneration. However, more studies are needed to confirm the safety and efficacy of these systems in humans and determine the optimal conditions for their use.

## 6. Conclusions

Nanosized lipid-based drug delivery systems have shown promise in the wound treatment challenges such as the low bioavailability of active drugs with low solubility. They can improve the stability of medications used in wound healing therapy, leading to greater efficacy and fewer adverse effects than those seen with more traditional formulations. In addition, the physiochemical features of the most encapsulated medications discussed in this review showed an increase in the rate at which wounds heal. Improved wound healing can also be achieved through nanosized, lipid-based drug delivery systems, which boost medication concentrations in the treated skin area. However, more research into nanosized, lipid-based drug delivery systems is warranted to enhance the skin’s permeability for pharmacological substances, boost bioavailability, and better control drug release. This would equip clinicians with more potent forms of therapy for wound care.

## Figures and Tables

**Table 1:** Different types of hybrid lipids- and biodegradable polymer-based systems in drug delivery.

Combinations Systems	Lipid-Based Nanoparticle	Polymer Biodegradable	Drug Association	Biological/ Biomedical Benefits	Experimental Design	Ref
Encapsulation of biodegradable polymer-lipid and matrix- antibiotic doxycycline	Polymer-lipid encapsulation matrix	Polylactic-co-glycolic acid (PLGA)	Doxycycline	reducing implant-related infection protect against implant-related osteomyelitis protection from infection	in vitro, in vivo	[45]
Ursolic acid-loaded lipid core nanocapsules (LCNs)	LCNs s	Poly l-lactic acid (PLLA)	Ursolic acid	very high encapsulation efficiency improve the quality of the wound healing process avoid the skin aging changes decreased estrogen levels	in vivo	[46]
Co-encapsulation of NLCs containing chitosan or sodium alginate	NLCs	Chitosan	Tea tree oil	antimicrobial effects against susceptible and resistant strains of *s. Aureus* and *p. Aeruginosa* increased the nlc-induced fibroblasts migration potential effectiveness for the management of wounds.	in vitro	[47]
Antimicrobial implant-coating to prevent biomaterial-associated infections	Lipid Encapsulation	Poly lactic-co-glycolic acid (PLGA)	Antimicrobial Peptide op-145	high initial release rate effective antimicrobial activity against *s. Aureus* alternative for coatings releasing conventional antibiotics	in vitro, in vivo	[48]
Nanostructured lipid carrier (NLC) formulations	Nanostructured lipid carrier	Polyacrylic acid polymer	Thrombomodulin)	re-epithelialization formulations stable, easy to prepare gel form, more advantageous for topical application	in vitro, in vivo	[49]
Composite of nanofibrous membranes of PLGA/aloe vera containing LNPs	LNPs	Plga and aloe vera	N.A.	enhanced formulation regarding elasticity and thickness. a promising strategy for the treatment of chronic wound	in vitro, in vivo	[50]
Silver sulfadiazine SLNs	SLNs	Platelet lysate	Hydroxypropyl methyl cellulose (HPMC) or chitosan glutamate (Cs Glu)	protect fibroblasts against the cytotoxic effect of silver sulfadiazine lack of impairing the association of platelet lysate. compatible mechanical properties, hydration, and adhesion properties suitable for application on skin lesions.	in vitro, in vivo	[51]
Thymoquinone loaded chitosan-lecithin micelles	N.A.	Chitosan	Soya lecithin	enhanced delivery of poorly soluble drug potential for carrier system, chitosan, drug, and hydrogel promoting dermal regeneration and tissue repairing during the wound healing process.	in vitro, in vivo	[52]
Nanoemulsion gel of curcumin-loaded chitosan polymer-based	Nanoemulsion	Chitosan	Curcumin	optimized formulation the best candidate for the treatment of wound healing	in vitro, ex vivo, in vivo	[53]
A kolliphor-based gel containing neomycin sulfate loaded in SLNs	SLNs	N.A.	Neomycin sulfate	time- and cost-saving high-quality more regulated release enhanced antibacterial activity	ex vivo	[54]
Chitosan–hyaluronic acid composite sponge scaffold	LNPs	Chitosan–hyaluronic acid	Andrographolide	appropriate porosity swelling ratio controlled superior wound healing reduced scar formation	in vitro, in vivo	[55]
Curcumin-loaded sodium hyaluronate immobilized vesicles	Hyalurosomes	Polymer immobilized nanovesicles	Curcumin	permitting to reach better physico-chemical properties and biological performances improving efficiency and fast healing process. appropriate for cosmetic, pharmaceutical, and medical devices products.	in vitro, in vivo	[56]
Detection of model pathogenic wound biofilms as an intelligent hydrogel wound dressing	N.A.	Polydiacetylene polymer	N.A.	suitable for initial high-through put dressing evaluation. advanced infection-detecting dressing for wound care reduces unnecessary use of antibiotics	in vitro, in vivo	[57]
Topical fusidic acid drug delivery assisted chitosan and phospholipid	Phospholipid	Chitosan	Fusidic acid	suitable drug resistance stability and therapeutic efficacy	ex vivo, in vivo	[58]
High drug-loaded, curcumin solid lipid nanoparticle hydrogel	Solid lipid nanoparticle	Hydrogel	Curcumin	safe, stable, and autoclavable extended and controlled release of curcumin improved antibacterial activity. accelerate wound closure down regulation of the inflammatory response and oxidative stress expedited re-epithelialization, angiogenesis, and enhanced granulation tissue formation. promising, particularly for wound healing activity increase solubility, stability, and sustained release of curcumin	in vitro, in vivo	[59]
Silence tumor necrosis factor α by lipid nanoparticles	LNPs	Degradable lipidoid	N.A.	reduced tnfα expression in nondiabetic wounds alternative therapeutic to address chronic inflammation for biological irregularities endemic to the diabetic wound	in vitro, in vivo	[60]
Hibiscus rosa Sinensis extract-loaded SLNs	SLNs, Lipids glycerol monostearate	Carpool and peg for gel.	N.A.	an effective way to enhance the effectiveness and in vivo activity useful carriers for the delivery of herbal drugs	in vivo	[48]
Silver@curcumin nanoparticles and chitosan nanofibers electrospun	Silver nanoparticles	Chitosan	Curcumin	the increase in the release rate of curcumin more potent antibacterial activity compared to commercial AgNPs dressing higher water absorption capability in maintaining a moist environment improved bioactivity for promoting tissue regeneration	in vitro, in vivo	[61]
Enhance the immunostimulation properties of cationic lipid nanocarriers for nucleic acid delivery	Lipid nanocarriers	N.A.	Nucleic acid	Prevent the alteration of immune cell response to stimuli	in vitro, in vivo	[62]
Prunus spinosa extract loaded with biomimetic nanoparticles	LNPs	N.A.	N.A.	anti-inflammatory activity useful for the encapsulation	in vitro, in vivo	[63]
Semisolid dosage forms containing curcumin-ampicillin SLNs	SLNs	N.A.	Curcumin-ampicillin	improve wound healing process reduce treatment costs increase compliance of patients	in vitro, ex vivo, in vivo	[64]
Topical antibacterial gel loaded with cefadroxil SLNs	SLNs	N.A.	Cefadroxil	effective infectious treatment eradicating infection from lower skin layers enhanced epithelialization rate alternative topical formulation for safe and fast wound healing treatment	in vivo	[65]
LNCs for transdermal delivery of siRNA	Lipid-shelled nanocarriers (liposomes)	Distearoyl-sn-glycero-3-phosphocholine (dspc), 1,2-distearoyl-sn-glycero-3- Phosphoethanolamine-n-pamino (polyethyleneglycol)	siRNA	an improved level of transmission through the transdermis	in vitro, in vivo	[66]
Retinoic acid-loaded SLNs surrounded by chitosan	SLNs	Chitosan	All-trans retinoic acid	accelerated closure of the diabetic wounds reduced leukocytes infiltration and scar, improved collagen deposition	in vitro, in vivo	[67]
Caryocar Brasiliense oil-loaded polymeric LCNs s	LCNs s	Poly(ε-caprolactone)	Caryocar Brasiliense oil	propitious characteristics, being comfortable and adaptable to the several parts of the body, maintaining moisture in the wound bed dry, and protecting the peripheral area a promising alternative for wound treatments, avoiding losses and complications related to cutaneous lesions	in vitro, in vivo	[68]
Chamomile oil-loaded SLNs	SLNs	N.A.	Chamomile oil	accelerating the wound healing activity rate the high healing activity rate enhancing effect on the wound area contraction, increased the re-epithelization grade increased collagen deposition, increased tensile strength, increased the tgf-ß levels decreased the il-1ß levels and mmp-9 activity	in vitro, in vivo	[69]
Cryostructurates of collagen/lipid nanoparticle–curcumin	LNPs	Collagen	Curcumin	desirable features of curcumin-loaded collagen cryostructurates when recast by double-encapsulation technology potential for prolonged therapeutic effect slow down degradation rate and prolong structural persistence a good candidate for treating chronic wounds	in vitro	[70]
Liposomal dexamethasone–moxifloxacin nanoparticles combinations With collagen/gelatin/alginate hydrogel	Liposomal	Collagen/gelatin/alginate	Dexamethasone–moxifloxacin	inhibited pathogenic bacterial proliferation. inhibit pathogen microorganism growth improve corneal wound healing great novel formulation for drug releasing treatment potential for ophthalmological diseases in the future treatment	in vitro, in vivo	[71]

**Table 2:** Biodegradable polymeric systems and their features in the wound healing process.

	**Biodegradable Polymer**		**Synthesis Method**	**Advantages**	**physicochemical Properties**	**Formulations**	**Therapeutic and Biological Effects of Formulations Composed of Polymers**	**Ref**
**Natural polymers**	Polysaccharides	Alginate	internal and external gelation	- Bioadhesive - Non-toxic - Non-immunogenic - Non-irritant - Able to form a gel	High mechanical and chemical stability, controllable swelling properties	Hydrogel Film Sponge Wafer Foam Others	- Enhanced re-epithelialization - Improved formation of granulation tissue	[129,130]
		Cellulose	polymerization and polycondensation	- Non-toxic - Porous structure - Mechanically stable	Renewable, strength, high crystallinity, lightness and stiffness properties water insolubility	Hydrogel Film Sponge Others	- Absorb wound exudates - Maintain local moisture	[131,132]
		Chitosan	deacetylation of chitin	- Mucoadhesive - Non-toxic - Antibacterial property	Spin-ability, ability to form a film	Hydrogel Film Sponge Powder Others	- Prevent wound infections - Hemostatic property - Promote migration and proliferation of keratinocytes	[133,134,135]
		Dextran	reaction catalyzed by dextransucrase	- Non-toxic - Possesses modifiable functional groups - Clinically safe	Hydrophile, flexible	Hydrogel	- Increase angiogenic responses - Facilitate skin regeneration	[136,137]
		Hyaluronic acid	crosslinking virgin HA with divinyl sulfone (DVS) in sodium bis(2-ethylhex-yl)sulfosuccinate (AOT) re-verse mi-celle systems under basic conditions	- Non-toxic - One of the major components of EMC - Non-immunogenic	High viscosity, elasticity, high capacity of holding water	Hydrogel Film Sponge Nanofiber mat	- Induce hemostasis phase - Regulate the inflammation	[137,138]
	Proteins	Collagen	synthesis occurs in the cells of fibroblasts	- Non-toxic - One of the major components of EMC	High tensile strength, flexible, insoluble in water	Hydrogel Film Sponge Freeze-dried sheet	- Promote fibroblast proliferation - Induce dermal remodeling	[135,139]
		Gelatin	inverse miniemulsion	- Non-toxic - low antigenicity - Non-immunogenic	Flexible melting temperature close to body temperature	Hydrogel Film Sponge Membrane Nanofiber mat	- Hemostatic property - Maintain local moisture - Moderate water transmission rate	[140,141]
		Silk	several stages: degumming after the fibroin dissolved monomeric units and regenerated into nanoparticles	- Excellent mechanical property - Oxygen permeable - Blood compatible	High tensile strength, high crystallinity, elasticity, thermal regulation	Hydrogel Film Nanofiber mat	- Promote migration and proliferation of keratinocytes - Impose impacts on the secretion of ECM	[142]
**Semi-synthetic polymers**		Cellulose derivatives	derived from natural and plant sources	- Non-toxic - Highly hydrophilic - Non-immunogenic	Excellent polymerization, high mechanical strength, water holding capability	Hydrogel Film Sponge Nanofiber mat	- Absorb wound exudates - Maintain local moisture - Accelerate tissue repair	[143]
		PLGA	emulsification–evaporation with water immiscible solvents	- Non-toxic - Excellent mechanical property	Hydrophile, tunable physiochemical properties	Hydrogel Membrane Nanofiber mat Nanoparticle	- Able to release loaded agents in a sustained manner - Lactate (one of the byproducts of PLGA) shows beneficial effects in wound healing	[144,145]
		PCL	ring-opening reaction of ε-caprolactone on diethylene glycol, with the catalyst stannous octoate	- Easily processable - Slow rate of degradation	Excellent mechanical property, hydrophobic, semicrystalline, high thermal stability	Nanofiber mat Mesh		[146,147]

## Data Availability

Not applicable.
